# A Case of Gaucher Disease With Atypical Presentation Mimicking Chronic Recurrent Multifocal Osteomyelitis

**DOI:** 10.7759/cureus.77028

**Published:** 2025-01-06

**Authors:** Hend Abd El Baky, Richard D Thomas, Abdur Khan, Rabheh Abdul-Aziz

**Affiliations:** 1 Pediatrics, John R. Oishei Children's Hospital, Jacobs School of Medicine and Biomedical Sciences, University at Buffalo, Buffalo, USA; 2 Pediatric Radiology, John R. Oishei Children's Hospital, Jacobs School of Medicine and Biomedical Sciences, University at Buffalo, Buffalo, USA; 3 Pediatric Pathology, John R. Oishei Children's Hospital, Jacobs School of Medicine and Biomedical Sciences, University at Buffalo, Buffalo, USA; 4 Pediatric Rheumatology, John R. Oishei Children's Hospital, Jacobs School of Medicine and Biomedical Sciences, University at Buffalo, Buffalo, USA

**Keywords:** bone lesion, chronic recurrent multifocal osteomyelitis (crmo), deficiency in glucocerebrosidase, gaucher's disease, lysosomal storage disorder

## Abstract

We present the case of a nine-year-old male who presented with recurrent bony pain episodes since the age of four, requiring multiple hospital admissions. Initial workup showed anemia, thrombocytopenia, prolonged prothrombin time (PT), partial thromboplastin time (PTT), and elevated C-reactive protein (CRP) and erythrocyte sedimentation rate (ESR). The patient underwent an open biopsy of the right femur with irrigation and debridement that was inconclusive, but cultures were positive for methicillin-resistant Staphylococcus aureus (MRSA) and group G hemolytic streptococci that were treated with clindamycin. Differential diagnosis was broad with a negative workup for malignancy, chronic granulomatous disease, infectious causes, sarcoidosis, and vasculitis. Multiple MRIs were consistent with bilateral femur osteomyelitis, right proximal tibia, and right humerus osteomyelitis with negative cultures on subsequent admissions. The patient was diagnosed with chronic recurrent multifocal osteomyelitis (CRMO). He had clinical and laboratory improvement for two years on methotrexate. He was then tried on a short course of steroids and adalimumab for worsening symptoms. Bone biopsy was repeated, and findings were consistent with Gaucher disease (GD), which was confirmed on biochemical testing with low beta-glucosidase activity. He was started on enzyme replacement therapy with clinical and biochemical improvement.

## Introduction

Lysosomal storage diseases are diseases caused by mutations in genes encoding a lysosomal enzyme. The result is a defective functioning of lysosomes with a resultant accumulation of substrates in excess inside the cells of various organs. Enzyme testing is usually the initial diagnostic test, but genetic analysis of the gene mutations adds precision [1].

Gaucher’s disease (GD) is an autosomal recessive lysosomal storage disorder (LSD) due to mutations in GBA1, resulting in a deficiency of the enzyme glucocerebrosidase. It is a rare disease and affects approximately one in 50,000 to one in 100,000 people in the general population; however, prevalence in Ashkenazi Jews is higher (1:500-1000) [2]. It is classified into three types based on the type and severity of neurological involvement. Type I (non-neuropathic) can present at any age. Type II (acute neuropathic) is characterized by rapid neurological decline and generally presents perinatally or within the first year of life. Type III (chronic neuropathic) has a highly variable spectrum of associated neurological and non-neurological manifestations and has onset in early childhood [2]. The degree and severity of symptoms vary widely and can include splenomegaly (more than 90% of patients), anemia, thrombocytopenia, bleeding history, monoclonal gammopathy of undetermined significance, and bone manifestations. Therefore, establishing the initial diagnosis can be challenging due to overlap with other disorders [2].

Bone manifestations in GD can include osteonecrosis, bone pain crises, lytic lesions, osteoporosis (across the lifespan), pathological fractures, and the Erlenmeyer flask deformity. Bone manifestations typically appear in adolescence [3]. A bone crisis is typically painful and affects predominantly the pelvis and the lower extremities and rarely the upper extremities. While the basis of the bone pathology observed in GD is still only partially understood, it is estimated that up to 75% to 90% of patients diagnosed with GD will experience some bone findings throughout the course of their disease [4,5].

Chronic recurrent multifocal osteomyelitis (CRMO) is an inflammatory disorder of bones and is a disease of childhood. Typical symptoms include pain, swelling, and local skin changes. There is no set criteria for diagnosis being a rare disorder, and therefore, it is a diagnosis of exclusion. It is due to disturbed regulation of the innate immune system, resulting in immune cell infiltration of the bone and subsequent osteoclast differentiation and activation, osteolysis, and bone remodeling. Though bone biopsies usually remain sterile, lesions mimic infectious osteomyelitis in histology and on radiographs [6].

## Case presentation

The patient is a nine-year-old male with a past medical history of obstructive sleep apnea requiring tonsillectomy and epistaxis. He presented at the age of four with recurrent bony pains requiring multiple hospital admissions (Table 1). His first hospital admission was at the age of four when he presented with right knee pain. Workup showed elevated C-reactive protein (CRP) of 26.45 mg/L (normal reference range 0-12 mg/L), elevated erythrocyte sedimentation rate (ESR) of 26 mm/hr (normal reference range 0.20-10 mm/hr), and mild anemia (hemoglobin 10.1; normal reference range 12.5-16.1 g/dL). Left lower extremity X-ray showed a non-aggressive lytic lesion in the proximal tibial diaphysis and posterior distal femoral metaphysis. He was treated symptomatically with non-steroidal anti-inflammatory drugs (NSAIDs), and the pain resolved. 

**Table 1:** Summary of hospital admissions Labs normal reference range: Hb (12.5-16.1 g/dL), platelets (150-450 x10^9/L), ESR (0-10 mm/hr), CRP (0.20-10.00 mg/L), PT (11-15 sec), PTT (25-34 sec) Hb: Hemoglobin, PT: Prothrombin time, PTT: Partial thromboplastin time, CRP: C-reactive protein, ESR: Erythrocyte sedimentation rate, MRSA: Methicillin-resistant *Staphylococcus aureus, *CRMO: Chronic recurrent multifocal osteomyelitis, NSAIDs: Non-steroidal anti-inflammatory drugs

Parameters	Initial admission (age: four years)	Readmission 1 (age: five years)	Readmission 2 (age: five years)	Readmission 3 (age: five years)	Readmission 4 (age: six years)	Readmission 5 (age: eight years)
Presentation	Right leg pain and inability to bear weight	Postoperative bleeding and respiratory distress following tonsillectomy and adenoidectomy	Right knee pain	Pain in the left popliteal fossa	Right leg pain	Right arm pain
Labs	Hb 10.1, CRP 26, ESR 52	Hb 8, platelets 102, PT 18, PTT 43	Hb 10, platelets 138, CRP 78, ESR 87, culture: MRSA and group G strep	Hb 9, platelets 132, PTT 47, CRP 15, ESR 43, cultures: negative	CRP 41, ESR 45	Hb 10, CRP 11.8, ESR 32
Imaging	X-ray: Non-aggressive lytic lesion in proximal tibial diaphysis		X-ray: Extensive lytic lesions in the proximal tibia and femur; MRI: large sub-periosteal fluid collection on the posterior aspect of the distal femur with extensive marrow signal abnormality and enhancing edema surrounding muscles	MRI: Extensive changes consistent with osteomyelitis of distal femur bilaterally with abscess; Echo: Bicuspid aortic valve	MRI: Findings consistent with right tibial osteomyelitis	MRI: Extensive intramedullary edema, periostitis, muscle edema
Management	Symptomatic treatment with NSAIDs	Hematology evaluation	Open biopsy of right femur; debridement and irrigation; clindamycin	Ceftriaxone and clindamycin	Incision, drainage, and irrigation of right proximal tibia; diagnosed with CRMO; methotrexate	Naproxen solu-medrol, prednisone, Humira; Repeat bone biopsy

**Figure 1:**
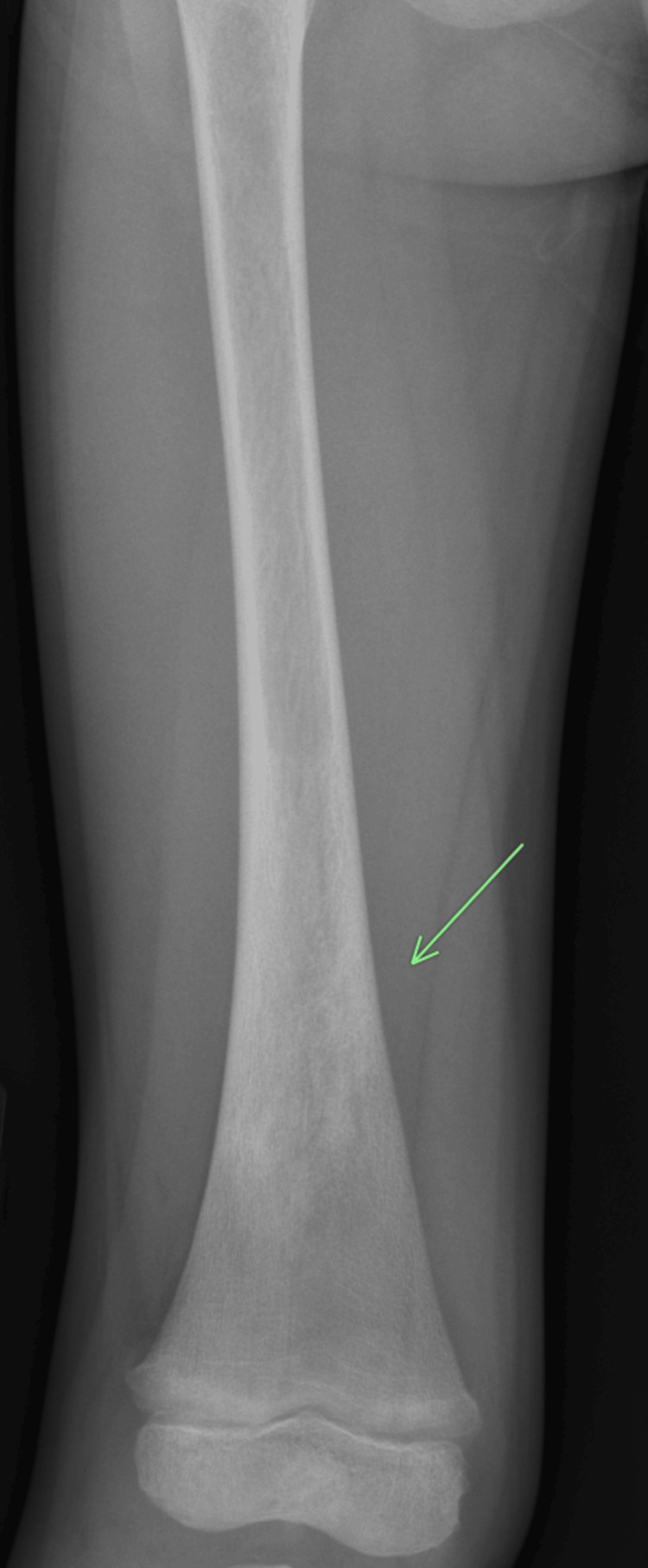
Anteroposterior X-ray distal right femur Seen is a heterogeneous lytic and sclerotic region in the distal diaphysis (oblique arrow).

He was readmitted one year later for right knee pain. Workup was significant for mild anemia (hemoglobin 10), mildly low platelet count of 138,000, elevated CRP of 78, and ESR of 87. The X-ray again showed increasingly extensive lytic lesions in the proximal tibia and femur that appeared non-aggressive (Figure 1). 

An MRI of the right femur was obtained and showed a large sub-periosteal fluid collection on the posterior distal femur with extensive marrow signal abnormality and ill-defined enhancing edema surrounding musculature. No effusion or synovial enhancement was seen. Findings were atypical for osteomyelitis. An open biopsy from the right femur with irrigation debridement was done after the patient was started on antibiotics. Histopathology was negative for malignancy and showed diffuse medullary changes in the femur without discernible inflammation suggestive of treated osteomyelitis. Fluid cultures were positive for methicillin-resistant *Staphylococcus aureus* (MRSA) and group G hemolytic streptococci; the patient was discharged home on clindamycin. The next month the patient developed left popliteal fossa pain and was re-admitted.

**Figure 2:**
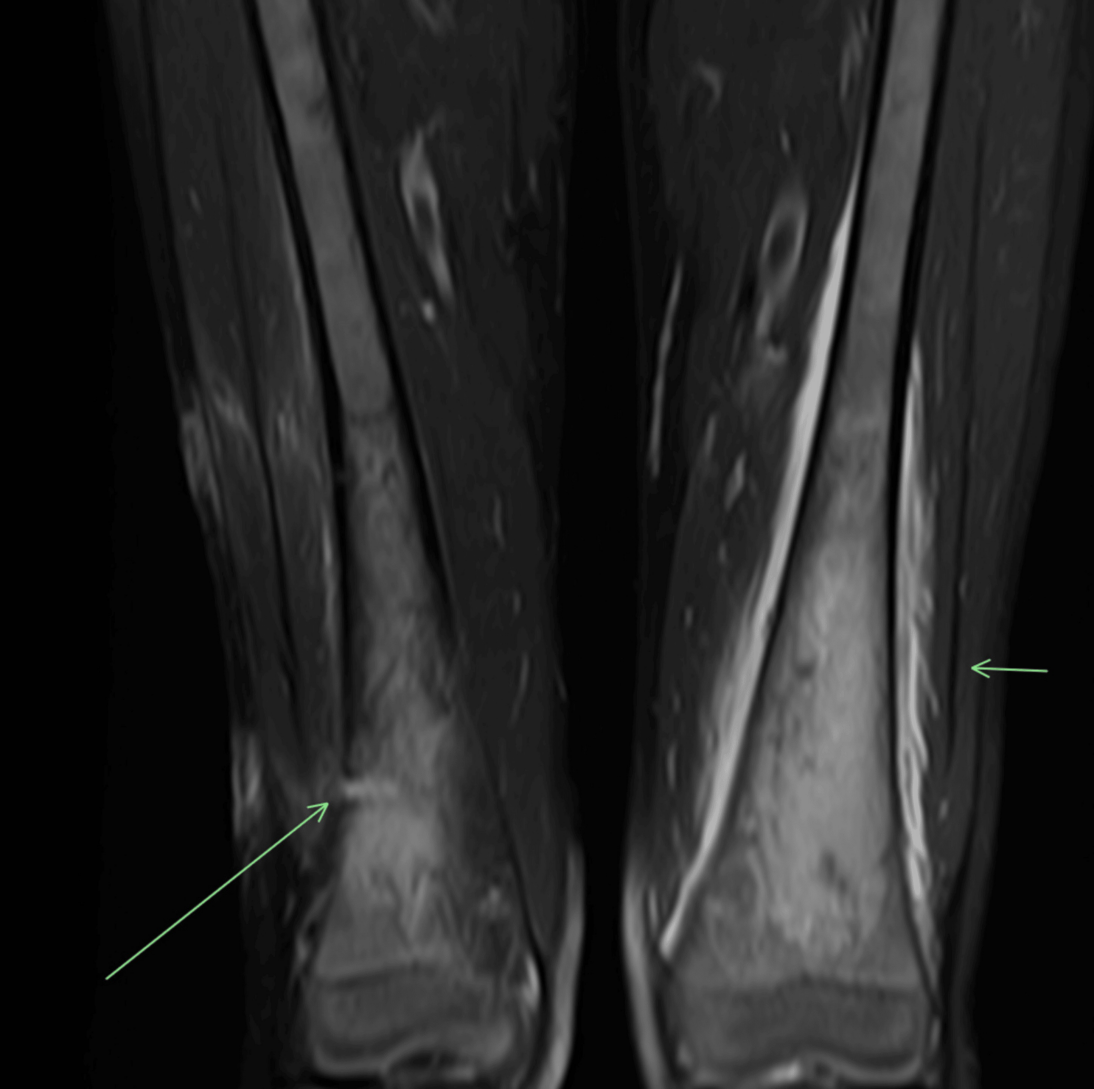
The 1.5T MRI coronal fat-saturated T2-weighted image of the bilateral femora Extensive marrow edema is noted in the left femoral metadiaphysis with subperiosteal fluid and paraosseous myositis (short horizontal arrow). Residual finding of marrow edema is observed in the right femoral metaphysis, with a lateral biopsy tract (long oblique arrow) and no significant extraosseous findings.

Workup for infectious causes included Lyme antibody testing, HIV antigen and antibody screen, hepatitis B panel, hepatitis C polymerase chain reaction (PCR), Quantiferon, *Histoplasma *urine antigen, and *Blastomyces* antibody, which were all negative. Workup for immunodeficiency disorders showed normal lymphocyte subsets, immunoglobulin levels, negative chronic granulomatous disease (CGD) with dihydrorhodamine (DHR) flow cytometry assay, negative C-antineutrophil cytoplasmic antibodies (ANCA) and P-ANCA as well as a negative workup for sarcoidosis. Repeat MRI of the femur showed extensive changes consistent with osteomyelitis of the distal femur bilaterally with a large posterior sub-periosteal abscess and a smaller lateral sub-periosteal abscess at the posterior aspect of the distal right femur with probable pus in the distal diaphysis and metaphysis of the left and right femur (Figure 2).

**Figure 3:**
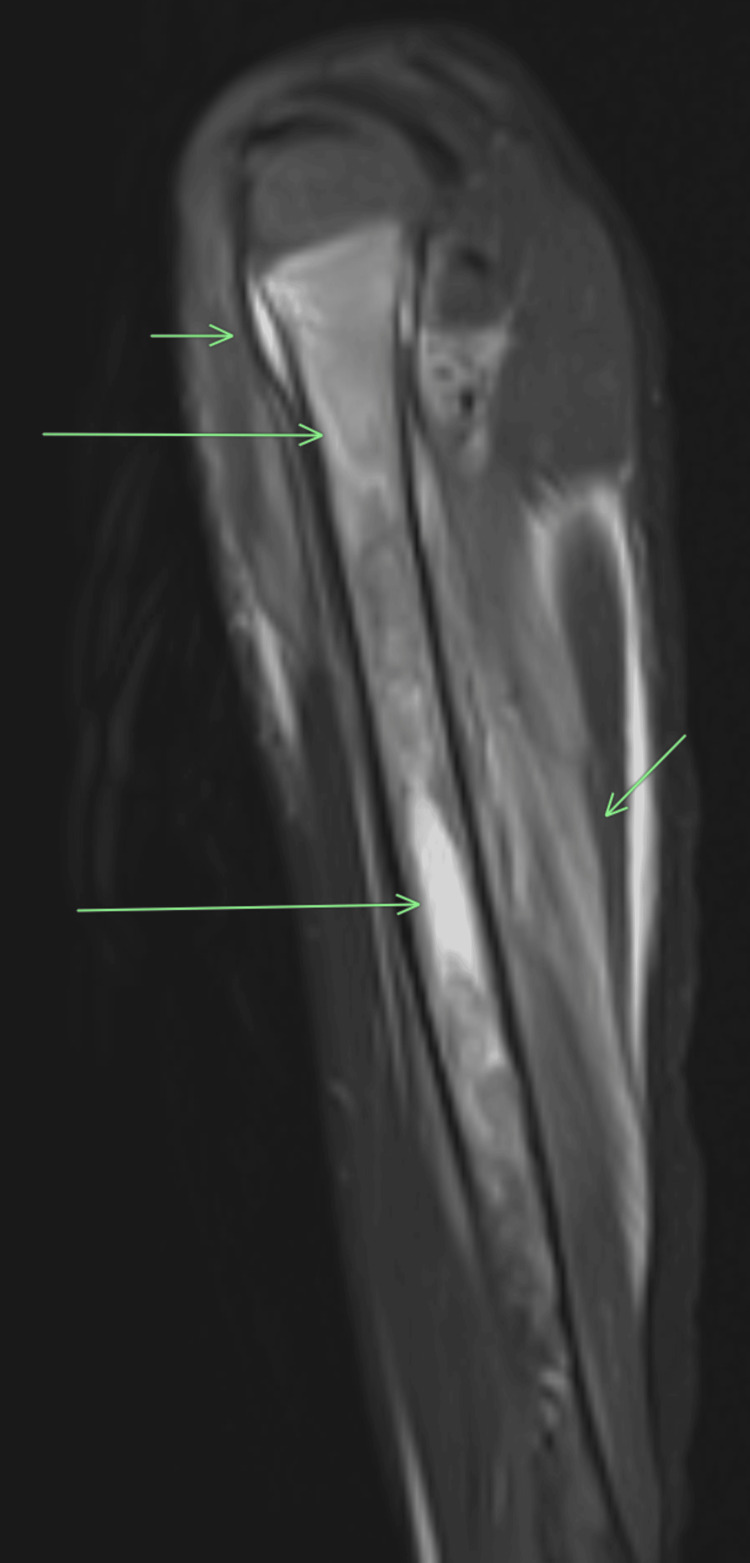
The 1.5T MRI sagittal T2 Dixon water image of the right humerus Extensive marrow edema is observed in the metadiaphysis (two long horizontal arrows) with subperiosteal fluid at the metaphysis (short horizontal arrow), and paraosseous myositis (short oblique arrow).

The patient was treated with ceftriaxone and clindamycin. Cultures from the left femur hemovac drain were negative. He was continued on clindamycin for six weeks and followed up by the orthopedics department. However, he was readmitted a year later for recurrent right leg pain and inability to bear weight. Again, MRI findings were consistent with right tibial osteomyelitis but with negative cultures. Rheumatology was consulted, and he was diagnosed with CRMO. He was started on methotrexate with clinical and biochemical improvement that lasted for almost 22 months. The patient developed right arm pain requiring hospital admission. An MRI of the upper extremity showed extensive intramedullary edema at the proximal humerus extending into the distal humeral metaphysis with periostitis (Figure 3). 

The patient was evaluated by the orthopedics and infectious diseases department; both felt that infectious osteomyelitis was unlikely. He was started on NSAIDs with significant improvement of his pain, and he also received a dose of IV methylprednisolone 30 mg/kg infusion and was discharged on a 10-day course of oral steroids. The decision was made to start the patient on adalimumab for better control of CRMO, which he tolerated well. His platelet counts were normalized. 

**Figure 4:**
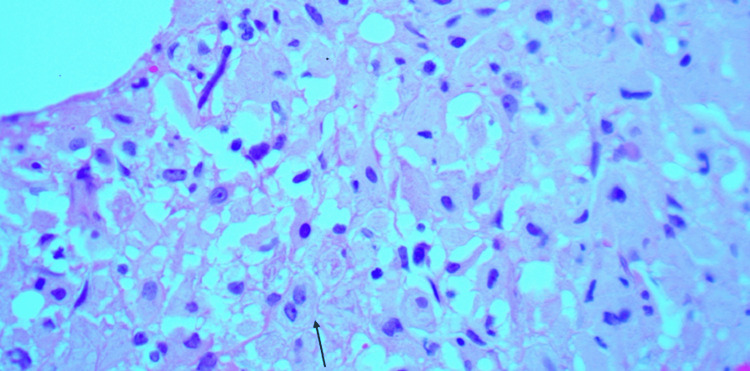
Bone biopsy showing numerous large aggregates of large histiocytic cells with copious eosinophilic cytoplasm characteristic of Gaucher disease (arrow)

The patient was readmitted again six months later for similar complaints. A decision was made to repeat the bone biopsy by orthopedic surgery for a better bone sample, as the previous biopsy was done by interventional radiology. The bone biopsy of the right distal femur showed morphologic findings characteristic of GD: numerous large aggregates of large histiocytic cells with copious eosinophilic cytoplasm (Figure 4).

Biochemical confirmation was done with low beta-glucosidase enzyme activity 0.74 nmol/h/mg (normal reference range ≥ 3.53 nmol/h/mg) and high glucopsychosine 0.359 nmol/ml (normal reference range ≤ 0.003 nmol/ml). Genetic testing for GBA gene sequencing confirmed the diagnosis of GD. Abdominal ultrasound showed normal spleen size. A decision was made to proceed with enzyme replacement therapy (velaglucerase alfa) with marked clinical and biochemical improvement that has been maintained for 24 months.

## Discussion

Type I GD is associated with episodes of bone crises that usually occur in the first two decades of life. It usually presents with acute onset of bone pain [7]. Pain can last from a few days to several weeks [8]. It can be severe enough to require the use of narcotics [9].

On MRI, high intramedullary and sub-periosteal signals can be seen on both T1 and T2 weighted sequences, suggesting a subacute hemorrhage or hematoma [7]. Reactive edema can be seen in surrounding muscles [7]. Osteonecrosis with areas of low and high bone density can be seen at the site of bone crisis a few months later [7]. Pathological fractures may occur as a complication. 

The pathogenesis of bone crisis in type I GD is unclear. Some investigators suggest that an increase in the intraosseous pressure may play a role given the immediate relief of pain after open bone decompression surgery [7]. In 1965 Yosipovitch et al. reported a patient who had decompression of the periosteum, which was distended by blood-stained fluid, after which he had immediate relief of pain. This patient had GD and was initially mistakenly diagnosed with osteomyelitis [8]. Increased microvascular permeability leading to fluid extravasation occurs in several pathophysiological conditions such as inflammation and sepsis. Treatment options for GD with bone manifestations or crisis include high-dose methylprednisolone [7]. Enzyme replacement therapy has been reported to prevent the progression of symptomatic skeletal disease. Bisphosphonates can reduce bone crises and fractures in patients of this type [10]. Despite being on enzyme replacement therapy, some patients can initially continue to have such episodes [10].

## Conclusions

Our patient had an atypical presentation of GD, i.e., bone pain without bruising or hepatosplenomegaly. The initial bone biopsy was not diagnostic, and as thrombocytopenia and prolonged PT in our patient cannot be explained by CRMO, the bone biopsy was repeated, which then confirmed the diagnosis. Gaucher disease should be considered in the differential diagnosis of patients presenting with recurrent bony pains with multifocal bony involvement.
